# Proposed algorithm during COVID-19 pandemic for patient management in medical retina clinic

**DOI:** 10.1186/s40942-020-00226-z

**Published:** 2020-06-03

**Authors:** Paolo Corazza, Francesco Maria D’Alterio, Saad Younis

**Affiliations:** Western Eye Hospital, Imperial College Healthcare NHS Trust, 171 Marylebone Rd, NW1 5QH London, UK

## Background

Over the last few months, the outbreak of coronavirus disease 19 (COVID-19) has affected an increasing number of Countries all over the world, and the United Kingdom (UK) is one of the most hit nations in Europe. The severe acute respiratory syndrome coronavirus 2 (SARS–CoV- 2), causing COVID-19, is thought to be transmitted through droplets, fomites, fecal material, and tears [1–3].

The absence of validated therapies and a vaccine has forced governments of many nations to implement lockdown and to apply the rules of social distancing (at least 1–2 meters between people). The detection of SARS-CoV-2 in tears and conjunctival secretions of infected patients with conjunctivitis and the short distance between patient and ophthalmologist during eye examinations and procedures, put eye doctors in high risk of being infected although not involved in the frontline. American Academy of Ophthalmology (AAO) on its website updated on a daily basis the recommendations to increase the protection of ophthalmic team members and patients during any visit, and highlighted some eye conditions that should be always monitored and treated [4]. In addition, many macular and retinal sight-threatening diseases require non-deferrable treatments that usually are performed in patients with systemic underling conditions (i.e. old age, diabetes, autoimmune diseases). This makes these patients at high risk for COVID-19. Royal college of ophthalmologists proposed guidelines on how to proceed specifically in Medical Retina (MR) clinic [5]. The World Health Organization (WHO) gave us technical guidance of the clinical management and important precautions that need to be set up during a pandemic [6]. Many reports suggested keeping patients at high risk of vision loss under defined treatment schemes [7–9]. Initially we reviewed the measures taken by health systems in Singapore and Hong Kong as published by Wong et al. [10] and by Lai [11]. We tried to match these guidelines in order to offer the best care possible to our patients. The purpose of this paper is to describe how the MR clinic at the Western Eye Hospital, Imperial college NHS trust in London, faced the COVID 19 pandemic. Our MR service is a tertiary referral centre for degenerative and vascular retinal diseases (such as age related macular degeneration (AMD), diabetic macular oedema (DMO), retinal vascular occlusion (RVO)) and other retinal disorders. During the 2019, in our clinic we provided around 15,500 visits. In Table 1 data about our clinical activity are described with comparison between the lockdown period in the UK so far, compared with the same period of the last year. In particular, between the 23rd of March and the 3rd of May 2019, 2345 patients attended our MR clinic for face-to-face consultations with an average of 55,83 patients per day. Of these patients, 882 (37,6%) received intravitreal (IVT) injections and 60 (2,56%) retinal laser treatments. Our aim is to propose a possible algorithm in order to minimize the patients visit maximising their visual outcome. It is important to highlight the features of our clinical practice that helped us during this period:

- Our clinic is a one-step MR clinic. This means that we are able to provide intravitreal injections to patients on the same day of the ocular examination, without any delay.
- Intravitreal (IVT) injections are performed in dedicated clean rooms and not in theatres. This reduces the waiting time for patients in the hospital.
- For wet AMD patients we were currently using treat and extend (TEX) regime. During this emergency, this scheme of treatment maybe helped us to protect patients with macular condition probably more easily compared to pro-re-nata (PRN).
- We have a Virtual clinic service for stable patient that do not need to be seen in the “face-to-face” clinics.
- Electronic Medical Records (EMR) represents a very helpful tool for clinicians. They allow doctors to access clinical records easily and remotely if needed.

**Table 1:** Number of consultations intravitreal injections (IVT) and laser treatments done in the lockdown period so far, compared to the same period of the last year (2019)

	From the 23rd of march to the 3rd of may 2019	From the 23rd of march to the 3rd of may 2020
Face-to-face consultations	2345	510
IVT injections	882	456
Retinal laser procedures	60	12
Telephone consultations	–	1830

Face-to-face consultations including patients underwent treatment such as IVT or retinal laser procedures

## Measures taken during Covid-19 pandemic

The complexity of these days has been highlighted by the lack of data published in literature. Reports from national and international societies are at the moment the most important guidelines and following their indications, we categorized our patients in 3 main groups on the basis of the possible irreversible complications due to a long deferment of the treatment [4, 5, 7–9, 12]:

- High Risk Patients (HRP): including patients who need to be seen urgently, as suggested by some international ophthalmic societies [5, 7–9]. In this category we grouped patients affected by wet AMD or secondary macular choroidal neovascularization (CNV), only eye patients with any macular disorder, and patient with active proliferative diabetic retinopathy (R3) [12].
- Low risk patients (LRP): including patients who may need to receive ocular treatment (such as IVT injections or laser), but it can be deferred over time with lower risk of permanent eye damage compared to the HRP group. This group included patients affected by RVO, DMO or central serous chorioretinopathy (CSCR) (5).
- Non urgent Patients (NUP): including patients who usually do not require treatments such as affected by retinal dystrophies, choroidal nevus and hydroxychloroquine screening patients.

All our patients were contacted by the NHS England by means of text messages in order to cancel all the scheduled appointments. All doctors in the team went through the EMR on a daily basis of every expected patient. HRPs were contacted and, after a telephone triage (Table 2), were suggested to attend their scheduled appointments if no suspicious symptoms for COVID 19. Other scheduled patients were deferred and a telephone consultation carried out for each of them. Patient are asked to respond to some questions (Table 2) and, based on their previous notes and subjective evaluation of their vision, their appointment was rescheduled. The algorithm we propose is created in order to help to avoid permanent visual loss in HRPs as well as to reduce the waiting/exposure time in department with less interactions which help to protect our vulnerable patients. As suggested from international societies and from international literature, we took all the measures to reduce the risk of COVID 19 infection for our patients and staff [4, 10, 11]. These included social distancing of 1.5 m between patients in the waiting area, surgical masks for patients and disposable protective personal equipment (PPE) for all the members of the MR team (surgical masks, face shields, scrubs, aprons, and gloves). We also promoted hand hygiene and ensured regular environmental sanitation. For pragmatic exposition we will make another distinction between new patients and follow up patients. Regarding the clinical management, for both groups of patients RCOphth clinical guidelines have been followed [5], and also some suggestions from other international societies have been taken into account [7–9].

**Table 2:** Questions done for every phone consultation

Questions	Management
Did you travel outside UK in the last 3 months? If Yes, where about?	If yes and if in any high risk area, patient not allowed to come to the clinic
Do you think you get in contact with somebody that resulted positive for coronavirus?	If yes, patient not allowed to come to the clinic
Are you self-isolating?	If yes, patient not allowed to come to the clinic
Do you have any cough, fever or shortness of breath?	If yes, patient not allowed to come to the clinic
Did you notice any change in your vision? Did you note any distortion on your Amsler Chart?	If yes to both of the questions, patient asked to come to the clinic
For patients affected by wet AMD, are you keen to come to have the injection done?	If not, explain to the patient the possible risks of suspending the intravitreal injections

## New patient pathways

**Fig. 1:**
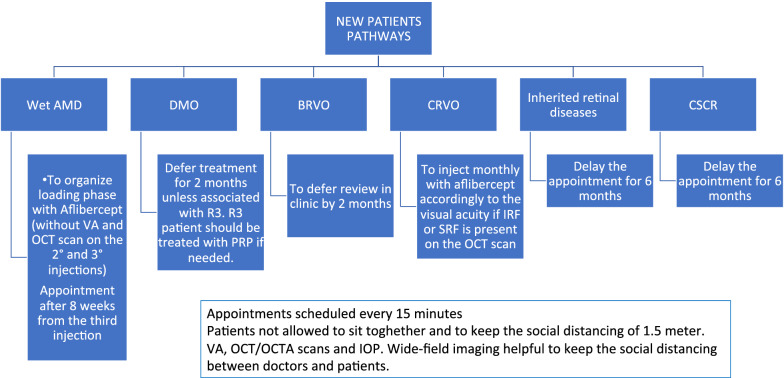
New patient pathway organized according to their retinal conditions

New patients can be referred to our clinic via the Accident and Emergency (A&E) or General Practitioners (GPs). Wet AMD patients who came in our clinic for the first time underwent: Best Corrected Visual Acuity (BCVA) evaluation, intraocular pressure (IOP) check, optical coherence tomography (OCT), OCT angiography (OCTA) and wide-field retinal imaging. Clinician then reviewed patients’ records and imaging and an Aflibercept IVT injection were administered in the clean room. In addition, other appointments were arranged for the second and the third IVT injections every 4 weeks (loading dose). Another appointment to see the clinicians were then scheduled after 8 weeks since the last injection. Patients affected by DMO referred to the clinic, were suggested to attend only if graded as R3 or only eye patients and were treated with panretinal photocoaugulation (PRP) laser and/or IVT Aflibercept injection if also DMO present. Every other diabetic patients needing IVT injection were deferred for 2 months. Although we considered patients affected from RVO as low priority, we evaluated patient with new diagnosed Central RVO (CRVO) in order to rule out an ischemic subtype needing urgent treatment, otherwise they were deferred for 2 months. In addition we started a monthly loading dose of Aflibercept in new CRVO patients if macular oedema present. Patient with Branch RVO (BRVO) were rescheduled after 2 months. Other patients affected by disease not needing an urgent treatment such as inherited retinal diseases or CSCR were rescheduled at least after 6 months. A summary chart is reported in Fig. 1.

## Follow up pathways

**Fig. 2:**
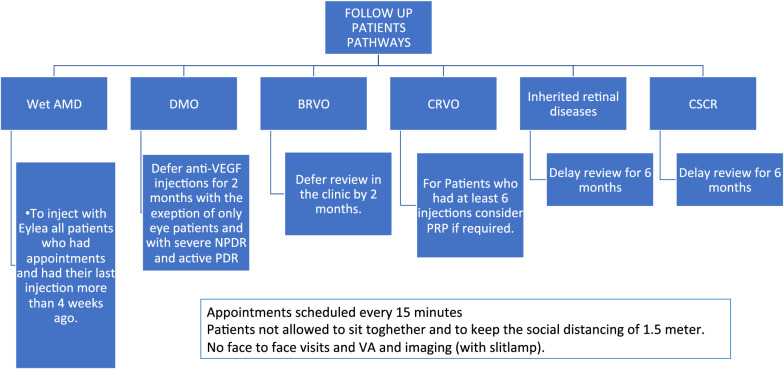
Follow up patient pathway organized according to their retinal conditions

We made telephone consultations for all the patients with a clinical appointment recorded. Appointment for LRPs were deffered and, instead, HRPs were invited to attend the clinic for treatment if no suspect COVID 19 symptoms detected during the telephone triage. For wet AMD or macular CNV patients we decided to inject with Aflibercept regardless to previous type of anti-vascular endothelial growth factor (VEGF) agents given because of its longer duration of action compared to other anti-VEGFs [13]. During the telephone triage we advised patients that no BCVA measurement, IOP check and imaging will be performed. We explained them that their appointment only included the administration of the treatment. Another follow up appointment was arranged after 2 months. All the patients who were not keen to come for the injections were advised of the possible risks of non-receiving IVT injection and, if still they did not want to come, we gave them another appointment in 4–8 weeks. They were also advised to attend our A&E department for any visual deterioration. Patients affected by DMO were not considered urgent and because of it the injections were deferred for 2 months. The only exceptions, as mentioned before, were R3 and only eyes patients affected by proliferative diabetic retinopathy that may benefit from retinal photocoagulation. Patients affected by RVO were rescheduled in 2 months if no record of new vessels on the disc or elsewhere or in the iris was recorded in their medical notes. For patients with CRVO complicated with chronic macula oedema PRP laser was considered if they already received at least 6 IVT injections. Patients affected by inherited retinal dystrophies and CSCR were called and booked for another appointment in at least 6 months. In Fig. 2 is reported the chart for follow up patients.

## Conclusions

Although telephone consultation has significant positive effects such as reassuring patients that emergency services are available for any visual deterioration or discussing their feeling during isolation, it presents also some issues. First of all, patients cannot be fully evaluated with imaging and functional tests, such as BCVA or IOP check, but we can only base our clinic decision on data reported by them. In addition, rarely this tool may present some communication difficulties in particular with patients with systemic underling conditions affecting their speech skills. Another factor to consider is the protection of patients’ data. Some measures need to be carried out in order to avoid sharing patient information to unauthorized persons: we always verify full name of the patient by phone, their date of birth and last attendance in our clinic before starting the consultation.

In general, our protocol did allow us to deliver the necessary treatment to the HRPs with a significant safety profile for patients and staff as it reduced the patients’ visit time to around 30 min. In addition, by means of telephone triage for COVID 19, we avoided that patient with suspect symptoms attending our clinic, as recommended by the Netherlands Ophthalmological Society [14]. In particular, 1830 patients received only telephone consultations, and 510 needed face-to-face consultation (average of 17 patients per typical clinical day). Of the last patients, the large majority received non-deferreable treatments: 456 (89,41%) IVT injections and 12 (2,35%) retinal lasers. No complications for patients receiving only telephone consultations or cases of patients infected by COVID 19 after attending our clinic have been reported so far.

Further improvement may be added to clinical practice in medical retina clinics, such as telemedicine arrangements and videophone consultations, but at the moment we think that this scheme can be used in many countries that are facing lockdown restrictions.

Our algorithm has some limitations: first of all the limited data available due to the short period of time during the lockdown, secondly the lack in literature available, especially in the beginning of the pandemic and, finally, the absence of evaluation of the outcomes that will need to be done after the end of the pandemic.

In conclusion, our protocol allowed our patients needing sight saving measures to be keep under a safe regimen scheme and avoided that patients with low/medium risk eye diseases would be exposed to COVID 19 infection.
